# Ferroptosis-dependent breast cancer cell-derived exosomes inhibit migration and invasion of breast cancer cells by suppressing M2 macrophage polarization

**DOI:** 10.7717/peerj.15060

**Published:** 2023-03-17

**Authors:** Chenghao Yi, Shilong Wu, Qianyu Duan, Lei Liu, Li Li, Yonghui Luo, Anan Wang

**Affiliations:** Department of Breast Surgery, The Second Affiliated Hospital of Nanchang University, Nanchang, The People’s Republic of China

**Keywords:** Breast cancer, Exosomes, M2 macrophage polarization, Ferroptosis

## Abstract

**Aim:**

Ferroptosis, a novel type of iron-dependent cell death, plays a vital role in breast cancer progression. However, the function of ferroptosis-induced cancer cell-derived exosomes in breast cancer remains unclear. In this study, we attempted to investigate the impact of breast cancer cells-derived exosomes induced by ferroptosis on the polarization of macrophages and the progression of breast cancer.

**Methods:**

Erastin was used to induce ferroptosis and breast cancer cell-derived exosomes were identified by transmission electron microscopy. Western blot, quantitative reverse transcription PCR, immunofluorescence, flow cytometry, and ELISA were used to determine the role of exosomes in macrophage polarization. Transwell assays were used to detect breast cancer cell migration, and invasion.

**Results:**

Our results showed that erastin promoted ferroptosis in breast cancer cells with increased Fe2+ level and ROS production. Breast cancer cell-derived exosomes induced by ferroptosis were successfully isolated and verified to be internalized by macrophages. In addition, ferroptosis-induced breast cancer cell-derived exosomes (Fe-exo) remarkably diminished M2 marker, Arg-1 expression. The ratio of CD206^+^ macrophages was significantly decreased after Fe-exo treatment. CD206 protein expression and Arg-1 level were dramatically reduced in M2 macrophages incubated by Fe-exo. Moreover, autophagy PCR array showed that the expression of 84 autophagy-related genes were altered after macrophages were incubated by Fe-exo. Furthermore, macrophages incubated by Fe-exo repressed the migration and invasion of breast cancer cells.

**Conclusion:**

Ferroptosis-dependent cancer cell-derived exosomes inhibited M2 polarization of macrophages, which in turn inhibited migration and invasion of breast cancer cells. This study provides novel therapeutic strategies for patients with breast cancer.

## Introduction

Breast cancer is the most common cancer in women and one of the leading causes of cancer death (Li, Mello-Thoms & Brennan, 2016). The incidence of breast cancer is the highest among all female malignancies and is increasing year by year worldwide. In 2020, 2,261,419 new breast cancer cases and 684,996 new breast cancer deaths were reported globally (Sung et al., 2021). Breast cancer is a heterogeneous disease, which has genetic and epigenetic changes as well as different subtypes and stages (Toss & Cristofanilli, 2015). The main treatment for breast cancer includes surgery, chemotherapy, and radiotherapy (Liyanage et al., 2019). Recently, advanced immunotherapy with potential for breast cancer treatment have emerged because of the potential of the immune system among a variety of novel therapeutic approaches. Although combination immunotherapy can improve the prognosis of breast cancer patients, the development of drug-resistance, metastases and tumor recurrence remains a huge challenge (Barzaman et al., 2021). Therefore, in order to prevent and treat breast cancer, it is particularly urgent to find novel effective drugs and therapeutic targets.

Ferroptosis is emerged as a new type of cell death, caused by iron-induced accumulation of lipid peroxides (Ding et al., 2021). Iron metabolism, lipid metabolism, ROS accumulation, cystine/glutamic acid reverse transporter (system Xc-), GSH, and GPX4 are involved in the occurrence of ferroptosis (Xie & Guo, 2021). In aspect of morphology, biology and genetics, ferroptosis is obviously different from other cell death, such as apoptosis, autophagy, necroptosis and pyroptosis (Mou et al., 2019). Increasing studies showed ferroptosis was involved in tumorigenesis, cancer development, metastases and resistances. In ovarian cancer, artesunate treatment increased the level of ROS in cells, leading to ferroptosis in ovarian cancer cells (Greenshields, Shepherd & Hoskin, 2017). Treating ovarian cancer cells with ferroptosis inhibitors reduced artesunate-mediated cytotoxicity. Resibufogenin suppressed colorectal cancer cell growth and tumorigenesis *via* inducing ferroptosis (Shen et al., 2021). At present, the role ferroptosis in breast cancer has attracted more and more attention. Wu et al. (2020) found that the GSK3 *β*/Nrf2 pathway regulated erastin-induced ferroptosis in breast cancer, and GSK3 *β* was expected to be an important target for enhancing erastin-mediated ferroptosis in breast cancer. Cao et al. (2022) discovered that curcumin repressed tumorigenesis by promoting ferroptosis in breast cancer. Thus, the induction of ferroptosis in tumor cells raises the prospect of its application in the treatment of breast cancer.

Exosomes are extracellular vesicles with a diameter of about 30-150nm and widely distributed in biological fluids and various cells (Liu et al., 2021b). Exosomes secreted by tumor cells can carry a variety of genetic materials of tumor cells, and act on vascular endothelial cells, tumor-associated fibroblasts, and immune cells in the tumor microenvironment to regulate tumor growth, metastasis, angiogenesis, and immune escape (Jia et al., 2017). A recent study reported that tumor-derived exosomes containing miR-34a inhibited migration and invasion of colorectal cancer cells (Hosseini et al., 2021). Another study discovered that p120-catenin-containing liver cancer cell-derived exosomes suppressed hepatocellular carcinoma cell proliferation and metastasis (Cheng et al., 2019). Breast cancer cell-derived exosomes, as important transfer carriers of intercellular communication and genetic material, are involved in breast cancer proliferation, invasion, metastasis, angiogenesis and immunosuppression by changing the signaling pathways, biochemical components and gene regulation of recipient cells (Wu et al., 2017; Xu, Yang & Lu, 2016). However, the relationship between ferroptosis-related breast cancer cell-derived exosomes and breast cancer cell progression remains largely unclear.

Macrophages are important immune cells in tumor microenvironment (Kwon et al., 2020). Macrophages can polarize into different states under different environments, and there are mainly two phenotypes, namely M1 and M2. Inhibition of M2 macrophage polarization can repress cancer progression and metastasis (Gu et al., 2022). For example, tumor cell-derived exosomal miR-770 suppressed the invasion of non-small cell lung cancer cells *via* inhibiting M2 macrophage polarization (Liu et al., 2021a). Hepatocellular carcinoma cell-derived exosomes with downregulated hsa_circ_00074854 inhibited M2 macrophage polarization to suppress migration and invasion in hepatocellular carcinoma cells (Wang et al., 2021). Cancer-derived exosomal miR-138-5p promoted M2 polarization in breast cancer (Xun et al., 2021). In addition, erastin-loaded exosomes induced ferroptosis and diminished the proliferation and migration of breast cancer cells (Yu et al., 2019). However, it remains unknown whether ferroptosis-induced breast cancer cell-derived exosomes regulate macrophage polarization to affect breast cancer progression.

In the present study, we aimed to explore whether breast cancer cell-derived exosomes induced by ferroptosis affect macrophage polarization. In addition, the role of macrophages incubated by ferroptosis-related breast cancer cell-derived exosomes in breast cancer cell progression was also delved into. These finding would shed new lights on exploring the novel strategies for breast cancer treatment.

## Materials and Methods

### Cell culture and polarization of M1 and M2 macrophages

Human acute monocytic leukemia cells, THP-1 cells, mouse macrophages, RAW 264.7 cells, human MDA-MB-231 cells, and mouse 4T1 cells were obtained from American Type Culture Collection (ATCC, Manassas, VA, USA). THP-1 cells, RAW 264.7 cells, human MDA-MB-231 cells, and mouse 4T1 cells were cultured in RPMI-1640 medium (Gibco, Waltham, MA, USA) containing 10% fetal bovine serum (Gibco, Waltham, MA, USA) at 37 °C in an incubator of 5% CO2 and 95% air. To induce THP-1 cells differentiate into macrophages, THP-1 cells were incubated with 100 ng/ml phorbol 12-myristate 13-acetate (PMA; Sigma-Aldrich) for 48 h. To obtain ferroptosis-induced breast cancer cells, MDA-MB-231 cells and 4T1 cells were cultured with ferroptosis inducer, erastin (10 µM) for 24 h.

PMA-induced THP-1 cells and RAW 264.7 cells were cultured with five ng/mL lipopolysaccharide (Sigma) and 100 U/mL IFN- *γ* (Sigma) for 24 h for M1 polarization. To obtain M2 macrophages, PMA-induced THP-1 cells and RAW 264.7 cells were cultured with 10 ng/mL IL-4 (Sigma) for 24 h.

### Cell Counting Kit-8 (CCK8) assay

The viability of MDA-MB-231 cells and 4T1 cells treated with erastin was evaluated *via* CCK-8 assay. Cells were seeded in 96-well plates with 2 × 10^3^ cells/well and cultured in a 5%CO2 incubator at 37 °C. CCK-8 solution (Dojindo Laboratories, Kumamoto, Japan) was added after culturing for 24, 48, or 72 h, and the OD 450 was measured by using a spectrophotometer (Infinite M1000; TECAN, Männedorf, Switzerland).

### Evaluation of reactive ferrous iron (Fe^2+^)

The Iron Colorimetric Assay Kit (#E1042; APPLYGEN) were used to detect the concentration of Fe^2+^ in the MDA-MB-231 cells and 4T1 cells treated with erastin. The absorbance of cells was detected by a spectrophotometer at 550 nm (Thermo Fisher Scientific, Waltham, MA, USA).

### Measurement of reactive oxygen species (ROS)

To detect the production of ROS in the MDA-MB-231 cells and 4T1 cells treated with erastin, cells were incubated in 10 µmol/L 2,7-dichlorofluorescein diacetate (DCFH-DA) (Sigma-Aldrich, St. Louis, MO, USA) in an incubator at 37 °C for 30 min. The sample was mixed upside down every 3-5 min so that the probe was in full contact with the cells. Then, cells were washed with serum-free cell culture medium for 3 times and 4′,6-diamidino-2-phenylindole (DAPI; Thermo Fisher) was used for nuclear staining. Laser confocal microscopy (Nikon, Tokyo, Japan) were used to observe the expression of ROS.

### Isolation and identification of exosomes

Exosomes were isolated from negative control (NC) or ferroptosis-induced MDA-MB-231 cells and 4T1 cells using ultracentrifugation. In brief, NC or ferroptosis-induced MDA-MB-231 cells and 4T1 cells were cultured in exosome-depleted medium for 72 h. The cell medium was gathered and centrifuged at 300 g at 4 °C for 10 min. Then media were filtered using a 0.22 µm filter. The media was ultracentrifuged at 120000 g for 70 min at 4 °C and exosomes were obtained. The exosome solution was resuspended in 30 µl of PBS. Whereafter, the isolated exosomes were detected by transmission electron microscopy (TEM) and specific exosome markers. Exosomes were fixed in 1% buffered glutaraldehyde for 10 min. The 20 µL of exosomes were added to formvar carbon-coated 300-mesh copper electron microscopy grids (Electron Microscopy Sciences, Hatfield, PA, USA), and permitted to stand for 5 min. Subsequently, exosomes were redyed using 2% uranyl oxalate at room temperature. After the grids were washed three times with PBS, the exosomes were analyzed using TEM (JEM-1200EX; TEM, Tokyo, Japan). Three positive markers (CD63, CD9, and Alix) and one negative marker (Fibronectin) were used for identification of exosomes *via* western blot analysis.

### Macrophages internalize breast cancer cell derived-exosomes induced by ferroptosis

NC or ferroptosis-induced MDA-MB-231 cell and 4T1 cell derived-exosomes were labelled with the fluorochrome DiI (Beyotime, Jiangsu, China) according to the manufacturer’s protocol. PMA-induced THP-1 cells and RAW 264.7 cells were incubated with DMEM containing corresponding DiI-labeled exosomes for 24 h. After washing the cells, DAPI were used to stain the cell nuclei. Finally, the results were analyzed under a fluorescence microscopy.

### Western blot (WB) analysis

The NC or ferroptosis-induced MDA-MB-231 cells and 4T1 cells, these cell derived-exosomes, and PMA-induced THP-1 cells and RAW 264.7 cells treated with these exosomes were lysed with RIPA. A protein assay kit was used to detect the concentration of total proteins. Equal protein (50 µg) was loaded on a 10% SDS gel and transferred to polyvinylidene fluoride membranes. Afterward, membranes were blocked in 5% milk in TBS-T for 2 h. Primary antibodies against CD63 (1:1000; Abcam, Cambridge, UK), CD9 (1:2000, Proteintech, USA), Alix (1:1000; Proteintech, Rosemont, IL, USA), Fibronectin (1:10000; Proteintech), IL-1β (1:1000; Santa Cruz, USA),

Arg-1 (1:1000; Proteintech), CD86 (1:1000; Invitrogen, Waltham, MA, USA), CD206 (1:1000; Abcam, UK) and GAPDH (1:10000; Proteintech) were utilized to incubate membranes at 4 °C overnight. Membranes were washed and incubated with goat anti-mouse IgG-HRP secondary antibody (1:1000; Beyotime) for 2 h. Enhanced chemiluminescence reagent was used to visualize the bands and the bands were analyzed by Image J.

### Quantitative reverse transcription polymerase chain reaction (qRT-PCR)

Total RNA was extracted from PMA-induced THP-1 cells and RAW 264.7 cells treated with corresponding NC or ferroptosis-induced MDA-MB-231 cell and 4T1 cell derived-exosomes using Trizol reagent (Invitrogen) and the concentration was measured by a Nanodrop ND-1000 spectrophotometer (Tiangen Biotech Co., Ltd., Beijing, China). Reverse transcription was performed with the Maxima First Strand cDNA Synthesis Kit (Promega, USA) for RT-PCR. qRT-PCR assay was performed using ABI QuantStudio 6 Flex system (Applied Biosystems, Foster City, CA, USA). The qRT-PCR reaction conditions were: 95 °C for 10 min, 40 cycles with 95 °C for 15 s and 60 °C for 60s followed by melting curves of the reaction. GAPDH was used as an internal reference gene. The relative mRNA expression levels of Arg-1, CD206, CD163, iNOS, and IL-1β were calculated by the 2^−ΔΔCt^ method. The sequences of all gene primers were shown in Table S1.

### Flow cytometry

PMA-induced THP-1 cells and RAW 264.7 cells were incubated with phycoerythrin (PE)-conjugated CD86 (Abcam, UK) and CD206 (Proteintech) at 4 °C for 30 min in the dark. Subsequently, cells were incubated with goat anti-rabit IgG-HRP secondary antibody (Abcam) for 30 min in the dark. Afterwards, flow cytometry analysis was performed in a FACSCalibur™ flow cytometer (BD Biosciences, Franklin Lakes, NJ, USA). Finally, the data were analyzed by Flowjo 7.6.1 (FlowJo, Ashland, OR, USA).

### Enzyme-linked immunosorbent assay (ELISA)

The level of Arg-1 in PMA-induced THP-1 cells and RAW 264.7 cells treated with corresponding NC or ferroptosis-induced MDA-MB-231 cell and 4T1 cell derived-exosomes was detected utilizing Arg-1 ELISA Kit in accordance with the manufacturer’s instructions.

### Immunofluorescence

PMA-induced THP-1 cells and RAW 264.7 cells treated with corresponding NC or ferroptosis-induced MDA-MB-231 cell and 4T1 cell derived-exosomes were seeded on cell slides and incubated overnight. Next, the cells were fixed in 4% paraformaldehyde at room temperature for 30min, permeabilized with 0.5% Triton X-100, and blocked with 1% bovine serum albumin (GIBCO, Waltham, MA, USA). The cells were incubated overnight at 4 °C with primary antibody against CD86 (1:100; Invitrogen) and Arg-1 (1:500; Proteintech). After washing with PBS, the cells were incubated with goat anti-mouse IgG-HRP secondary antibody (1:500, Abcam) for 1 h at room temperature. DAPI (Abcam) was used to stain cell nucleus. After washing 3 time with PBS, the sections were performed for photograph under a fluorescence microscope (Carl Zeiss, Oberkochen, Germany).

### Autophagy PCR array

RNA of PMA-induced THP-1 cells treated with NC or ferroptosis-induced MDA-MB-231 cell derived-exosomes was extracted using the RNeasy min kit (Qiagen, Hilden, Germany). cDNA was synthesized by using the RT2 First Strand kit (Qiagen). Afterwards, the PCR reaction was performed as follows: initial denaturation at 95 °C for 10 min, followed by 40 cycles of 95 °C for 15s and 60 °C for 1min. All samples were analyzed using the 2-ΔΔCt method with GAPDH as an internal control for normalization. The protein–protein network was constructed using Cytoscape V3.6.0.

### Transwell assay

PMA-induced THP-1 cells and RAW 264.7 cells treated with corresponding NC or ferroptosis-induced MDA-MB-231 cell and 4T1 cell derived-exosomes, and macrophage supernatants were collected. Subsequently, MDA-MB-231 cells and 4T1 cells were incubated with the supernatants. The migration and invasion of MDA-MB-231 cells and 4T1 cells were investigated using the Transwell system (Corning, NY, USA). For cell migration assay, breast cells were seeded in the upper chambers of 24-well Transwell plates with serum-free DMEM. DMEM with 10% FBS was then added into the lower chambers. After incubation for 24 h at 37 °C, cells were washed with PBS, fixed in 4% formaldehyde for 15 min at room temperature and stained using 1% crystal violet solution (Beyotime) for 20 min. Finally, migratory cells were counted under a microscope. To detect the invasion ability, breast cancer cells were seeded in Transwell plates pre-coated with Matrigel matrix (BD Biosciences) and cell invasion assay was performed according to the same experimental procedures as described above.

### Statistical analysis

Data were analyzed by SPSS 24.0 and presented as mean ± SD. *T*-test was used to analyze the difference between two groups, and one-way analysis of variance (ANOVA) was used to determine the statistical significance of more than two groups. *p* < 0.05 was considered statistically significant.

## Results

### Ferroptosis was induced by erastin in breast cancer cells

**Figure 1:**
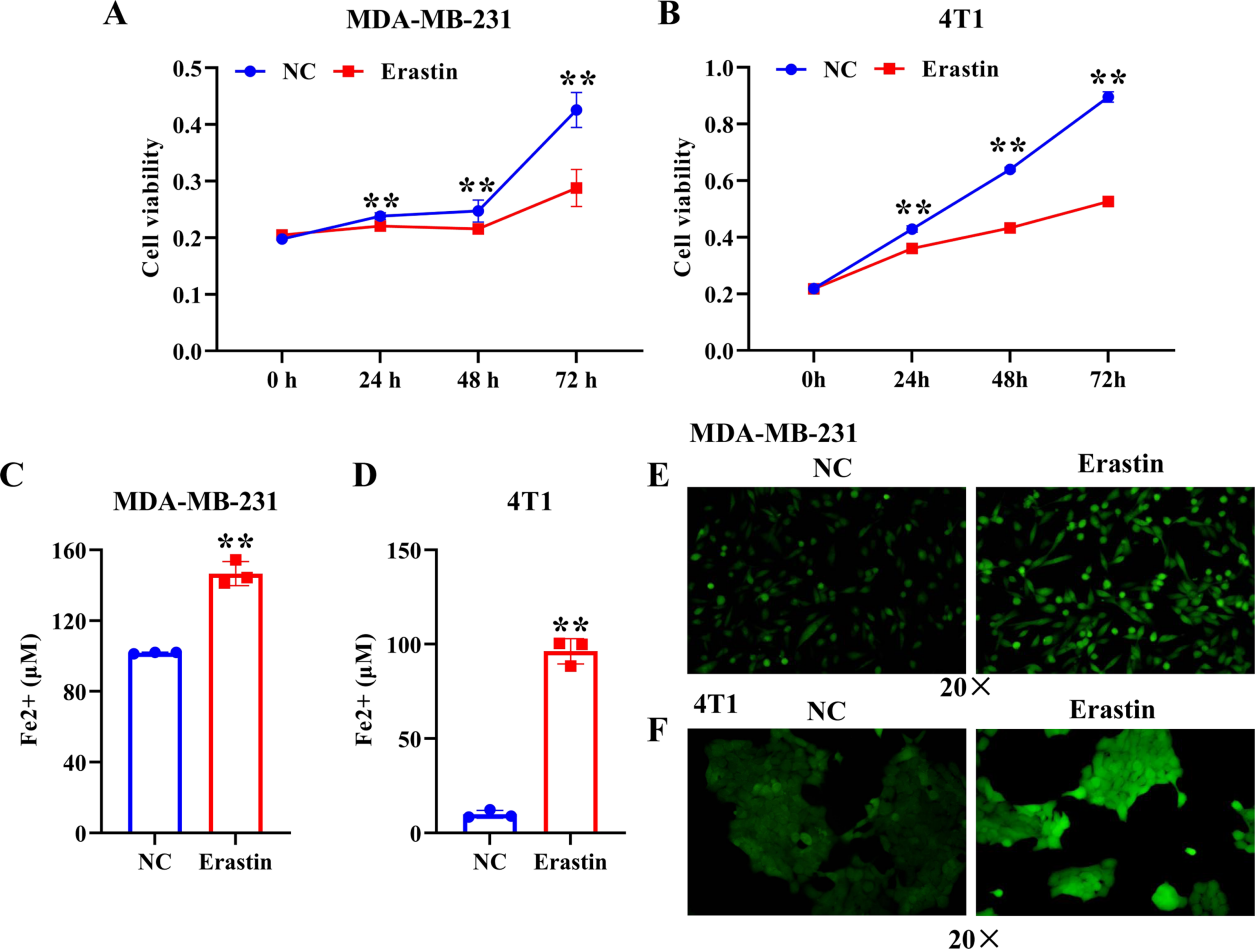
Erastin triggers ferroptosis in breast cancer cells. (A, B) Cell viability of MDA-MB-231 cells and 4T1 cells treated with erastin was measured by a CCK-8 assay. (C, D) The level of Fe2+ in MDA-MB-231 cells and 4T1 cells was determined by the iron assay kit. (E, F) Effect of erastin on the level of ROS in MDA-MB-231 cells and 4T1 cells. NC: negative control. The production of ROS was measured using DCFH-DA fluorescent probe. Magnification: 20 ×. Data are presented as the mean ± SD. ** *p* < 0.01.

To induce ferroptosis in MDA-MB-231 cells and 4T1 cells, we treated them with erastin, a ferroptosis inducer, and verified ferroptosis by CCK-8, iron assay kit, and immunofluorescence assay. The results showed that erastin resulted in a significant decrease of MDA-MB-231 cell and 4T1 cell viability compared with the NC group (Figs. 1A, 1B). The level of Fe2+ and ROS were dramatically enhanced in MDA-MB-231 cells and 4T1 cells induced by erastin relative to the NC group (Figs. 1C–1F). Collectively, ferroptosis was induced in breast cancer cells by using erastin.

### Macrophages internalize ferroptosis-induced breast cancer cell-derived exosomes

**Figure 2:**
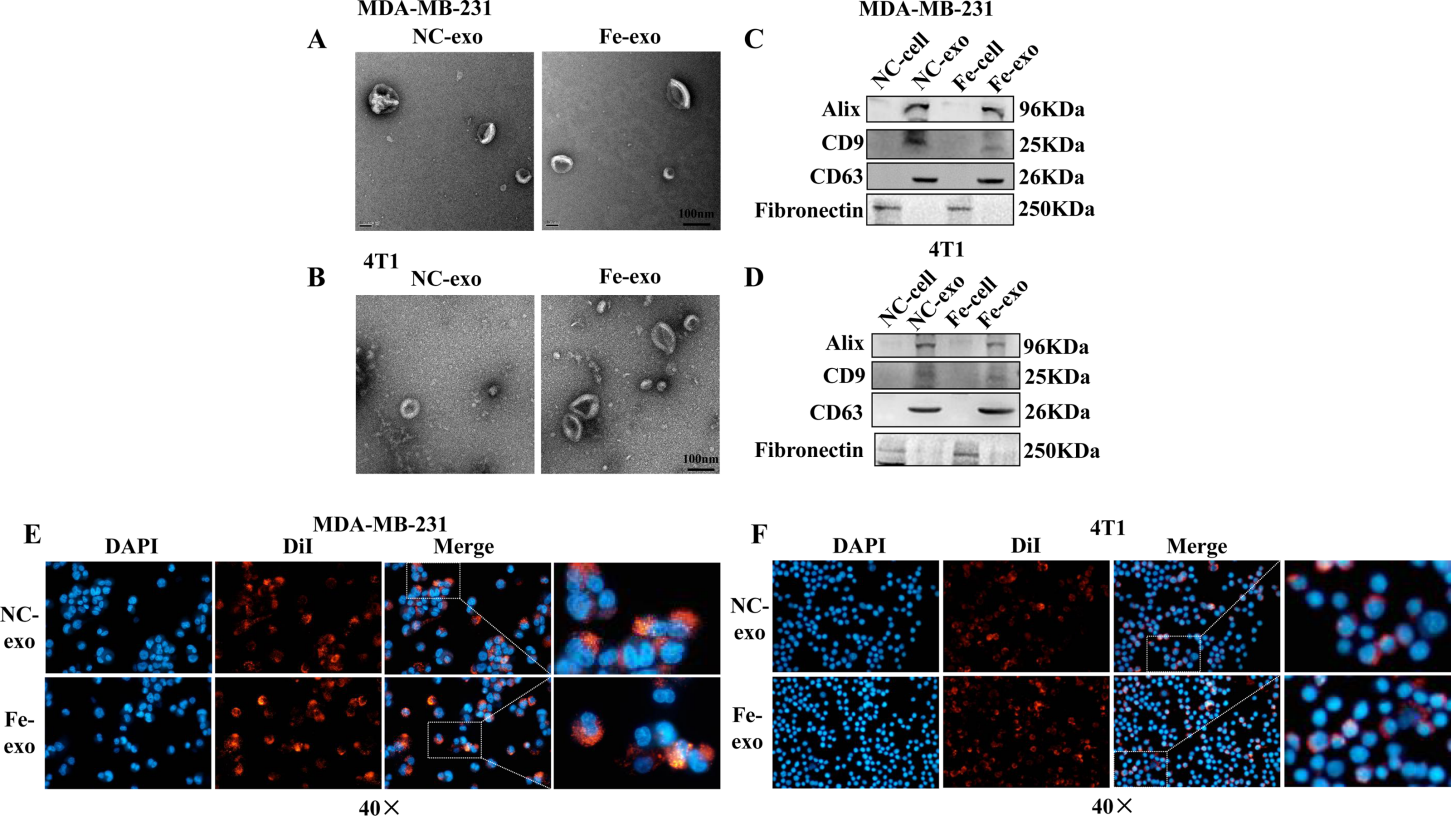
Macrophages internalize breast cancer cell-derived exosomes induced by ferroptosis. (A, B) Exosomes released by negative control (NC) or ferroptosis-induced MDA-MB-231 cells and 4T1 cells were evaluated using a transmission electron microscopy. Scale bars are 100 nm. (C, D) The protein expression of exosome markers was detected by western blot. The isolated exosomes from negative control (NC) or ferroptosis-induced MDA-MB-231 cells and 4T1 cells were identified by specific exosome markers. (E, F) MDA-MB-231 cell and 4T1 cell-derived exosomes internalization were observed under a laser scanning confocal microscope. Immunofluorescence was used to observe whether DiI-labeled exosomes from ferroptosis-induced MDA-MB-231 cells and 4T1 cells were taken up by PMA-induced THP-1 cells and RAW 264.7 cells, respectively. NC-cell represents negative control breast cancer cells. Fe-cell represents ferroptosis-induced breast cancer cells. NC-exo represents negative control breast cancer cell-derived exosomes. Fe-exo represents ferroptosis-induced breast cancer cell-derived exosomes. Magnification: 40 ×.

To investigate the role of exosomes produced by ferroptosis-mediated MDA-MB-231 cells and 4T1 cells, we first isolated exosomes from normal and ferroptosis-induced MDA-MB-231 cells and 4T1 cells. TEM images indicated that the morphology of both exosomes exhibited a near spherical shape with a diameter between 50 and 200 nm (Figs. 2A, 2B). WB showed that the protein expression of exosome markers, Alix, CD9, and CD63, was increased in NC and ferroptosis-induced MDA-MB-231 cell and 4T1 cell-derived exosomes compared with corresponding cancer cells (Figs. 2C, 2D). Fluorescence microscopy showed that red fluorescence was found in PMA-induced THP-1 cells and RAW 264.7 cells that had been incubated with MDA-MB-231 cell and 4T1 cell-derived exosomes induced by ferroptosis, indicating that exosomes were internalized by macrophages (Figs. 2E, 2F).

### Ferroptosis-induced cancer cell-derived exosomes inhibit M2 polarization of macrophage

**Figure 3:**
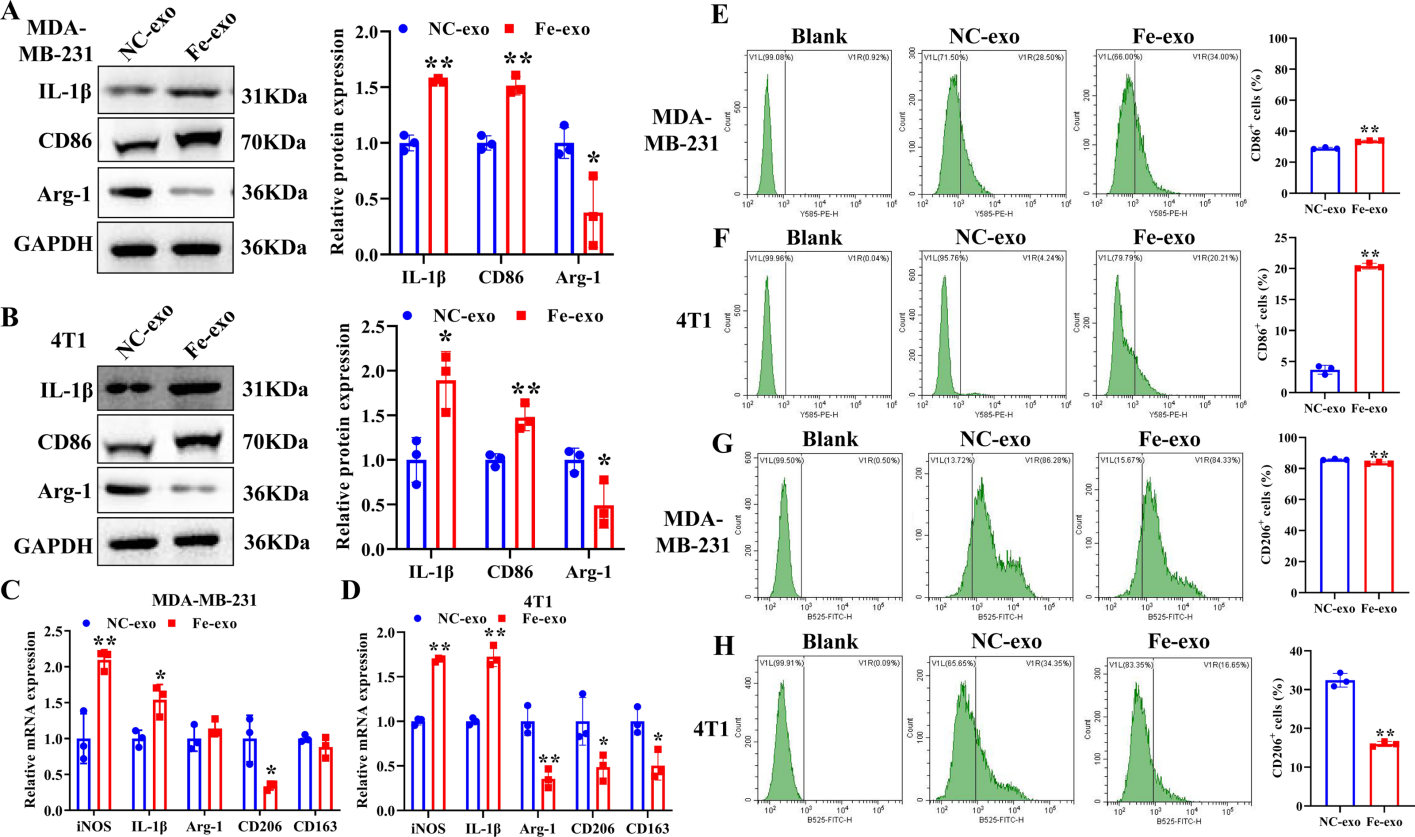
Effects of breast cell-derived exosomes induced by ferroptosis on macrophage polarization. (A, B) The protein expression of M1 and M2 macrophage markers were detected in PMA-induced THP-1 cells and RAW 264.7 cells incubated by negative control (NC) or ferroptosis-induced MDA-MB-231 and 4T1 cell-derived exosomes by western blot. (C, D) qRT-PCR was used to detect the mRNA expression of M1 and M2 macrophage markers in PMA-induced THP-1 cells and RAW 264.7 cells incubated by NC or Fe-induced MDA-MB-231 and 4T1 cell-derived exosomes. (E, F) Flow cytometric analysis of CD86-positive cells in PMA-induced THP-1 cells and RAW 264.7 cells. (G, H) Flow cytometric analysis of CD206-positive cells in PMA-induced THP-1 cells and RAW 264.7 cells. Macrophages were gated in accordance with the SSC/CD86 and SSC/CD206 dot plots. PMA-induced THP-1 cells were incubated by NC or Fe-induced MDA-MB-231 cell-derived exosomes. RAW 264.7 cells were incubated by NC or Fe-induced 4T1 cell-derived exosomes. NC-exo represents negative control breast cancer cell-derived exosomes. Fe-exo represents ferroptosis-induced breast cancer cell-derived exosomes. Data are presented as the mean ± SD. * *p* < 0.05. ** *p* < 0.01.

**Figure 4:**
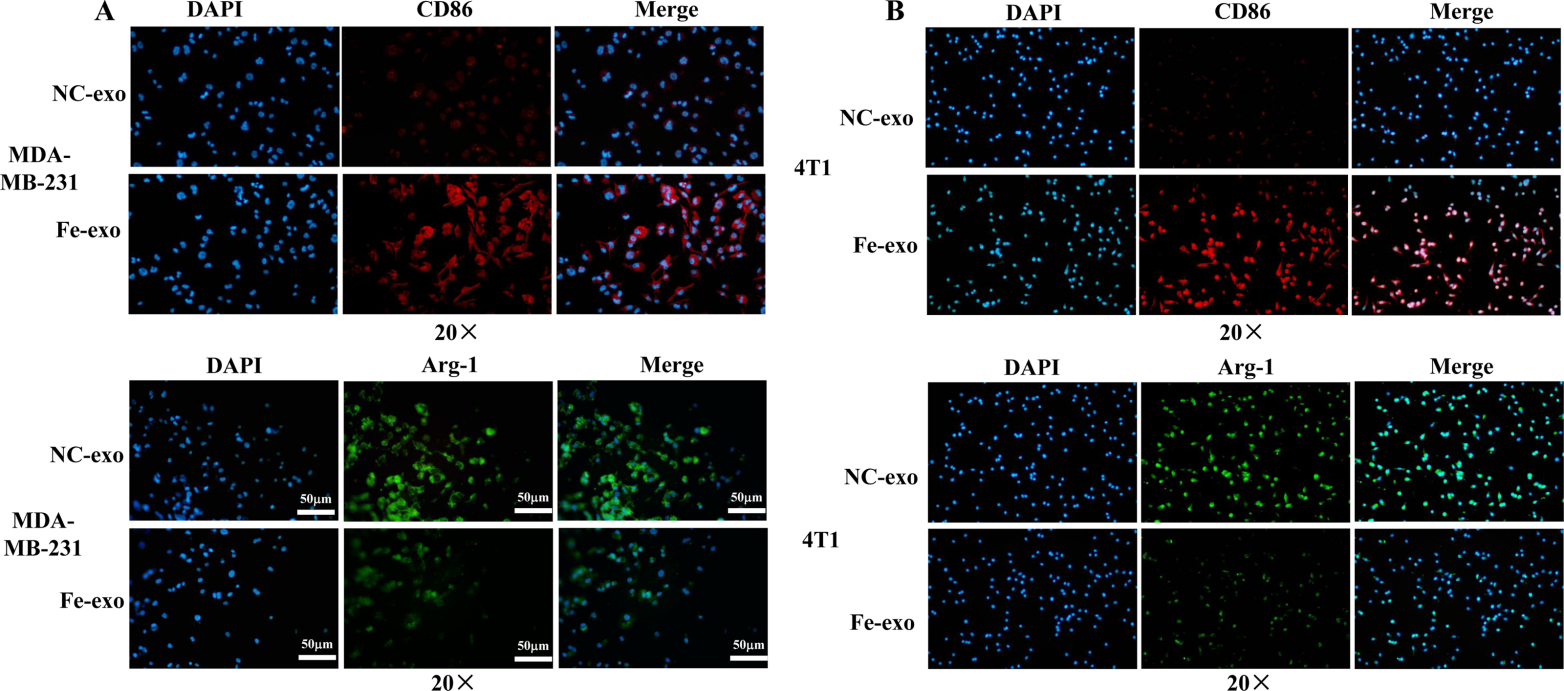
Effects of ferroptosis-induced breast cancer cell-derived exosomes on expression of M1 and M2 macrophage markers. (A) The expression of CD86 and Arg-1 in PMA-induced THP-1 cells incubated by NC or Fe-induced MDA-MB-231 cell-derived exosomes was measured by immunofluorescence. (B) The expression of CD86 and Arg-1 in RAW 264.7 cells incubated by NC or Fe-induced 4T1 cell-derived exosomes was measured by immunofluorescence. Magnification: 20 ×. NC-exo represents negative control breast cancer cell-derived exosomes. Fe-exo represents ferroptosis-induced breast cancer cell-derived exosomes.

**Figure 5:**
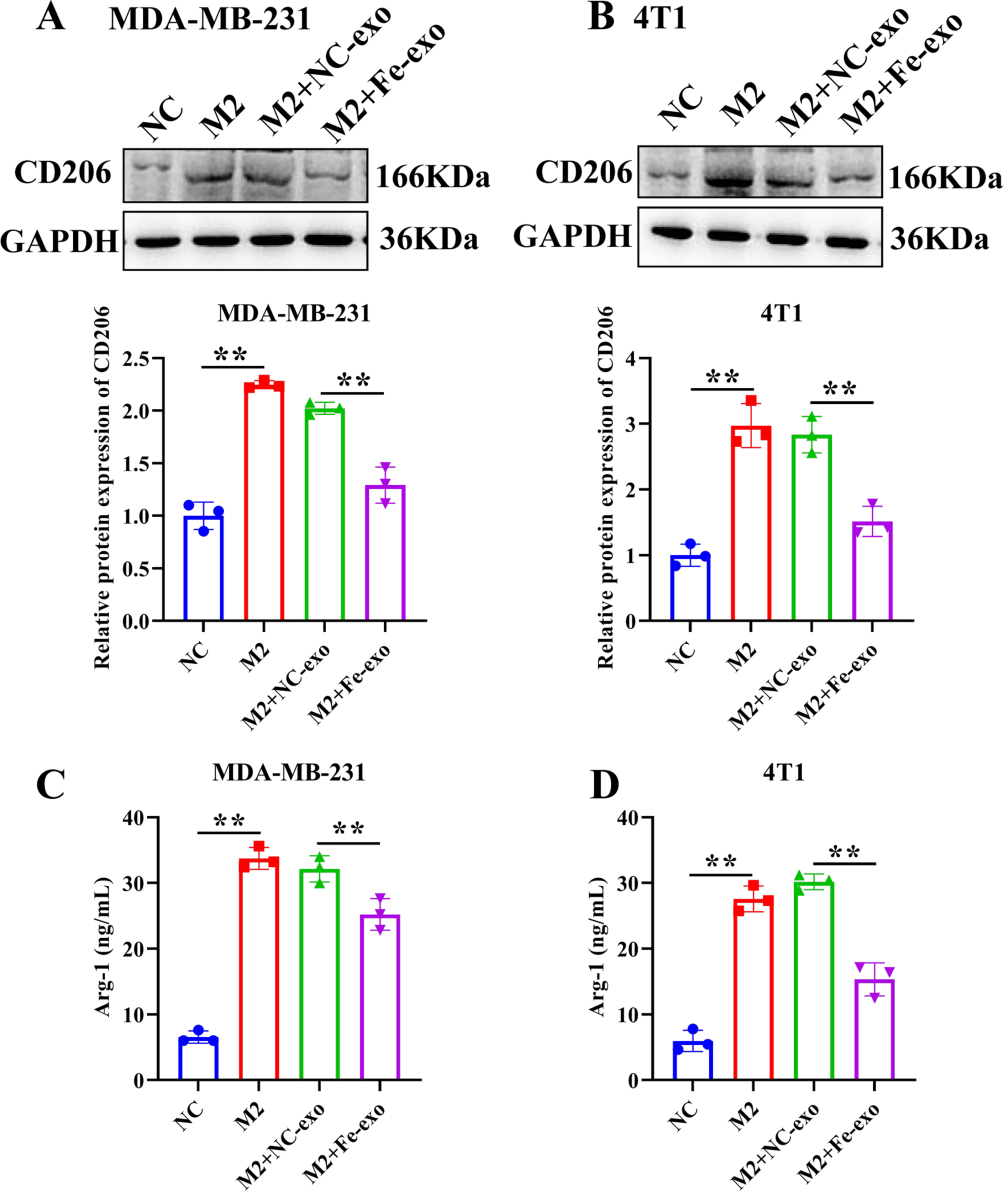
Effects of ferroptosis-induced breast cancer cell-derived exosomes on M2 macrophage polarization. (A, B) The protein expression of CD206 were detected in THP-1 and RAW 264.7 cell-derived M2 macrophages incubated by negative control (NC) or ferroptosis-induced MDA-MB-231 and 4T1 cell-derived exosomes by western blot. (C, D) The level of Arg-1 were detected in THP-1 and RAW 264.7 cell-derived M2 macrophages incubated by NC or Fe-induced MDA-MB-231 and 4T1 cell-derived exosomes by ELISA. Data are presented as the mean ± SD. * *p* < 0.05. ** *p* < 0.01.

To study the effect of breast cancer cell-derived exosomes induced by ferroptosis on the phenotype of macrophages, PMA-induced THP-1 cells and RAW 264.7 cells were incubated with erastin-treated MDA-MB-231 cell and 4T1 cell-derived exosomes, respectively. WB revealed that the protein expression of M2 marker, Arg-1 was dramatically reduced, while M1 markers, CD86 and IL-1β protein expression was significantly enhanced in macrophages incubated with exosomes derived from ferroptosis-induced MDA-MB-231 cells and 4T1 cells compared with negative control exosomes (Figs. 3A, 3B). In addition, qRT-PCR analysis showed that M2 markers, CD206 and CD163 mRNA expression was significantly decreased, while M1 markers, iNOS and IL-1 *β* mRNA expression was markedly increased in PMA-induced THP-1 cells and RAW 264.7 cells treated with MDA-MB-231 cell and 4T1 cell-derived exosomes induced by ferroptosis relative to macrophages treated with negative control exosomes (Figs. 3C, 3D). Flow cytometric analysis uncovered that the number of CD86^+^ cells was markedly enhanced, while CD206^+^ cells was obviously diminished in above macrophages incubated with ferroptosis-induced MDA-MB-231 cells and 4T1 cell-derived exosomes (Figs. 3E–3H, Fig. S1). The immunofluorescence test showed that the fluorescent expression of CD86 was remarkably increased, whereas Arg-1 was obviously reduced in above macrophages treated with ferroptosis-induced MDA-MB-231 cell and 4T1 cell-derived exosomes (Figs. 4A, 4B). In addition, the protein expression of CD206 was remarkably enhanced in M2 macrophages, while reduced in M2 macrophages incubated by ferroptosis-induced MDA-MB-231 cell and 4T1 cell-derived exosomes (Figs. 5A, 5B). ELISA results showed that M2 macrophages exhibited a significant increase in Arg-1 level, while M2 macrophages incubated by ferroptosis-induced MDA-MB-231 cell and 4T1 cell-derived exosomes exhibited an opposite result (Figs. 5C, 5D). These above data demonstrated that breast cancer cell-derived exosomes induced by ferroptosis inhibited polarization of M2 macrophages.

### Ferroptosis-induced cancer cell-derived exosomes regulate autophagy

**Figure 6:**
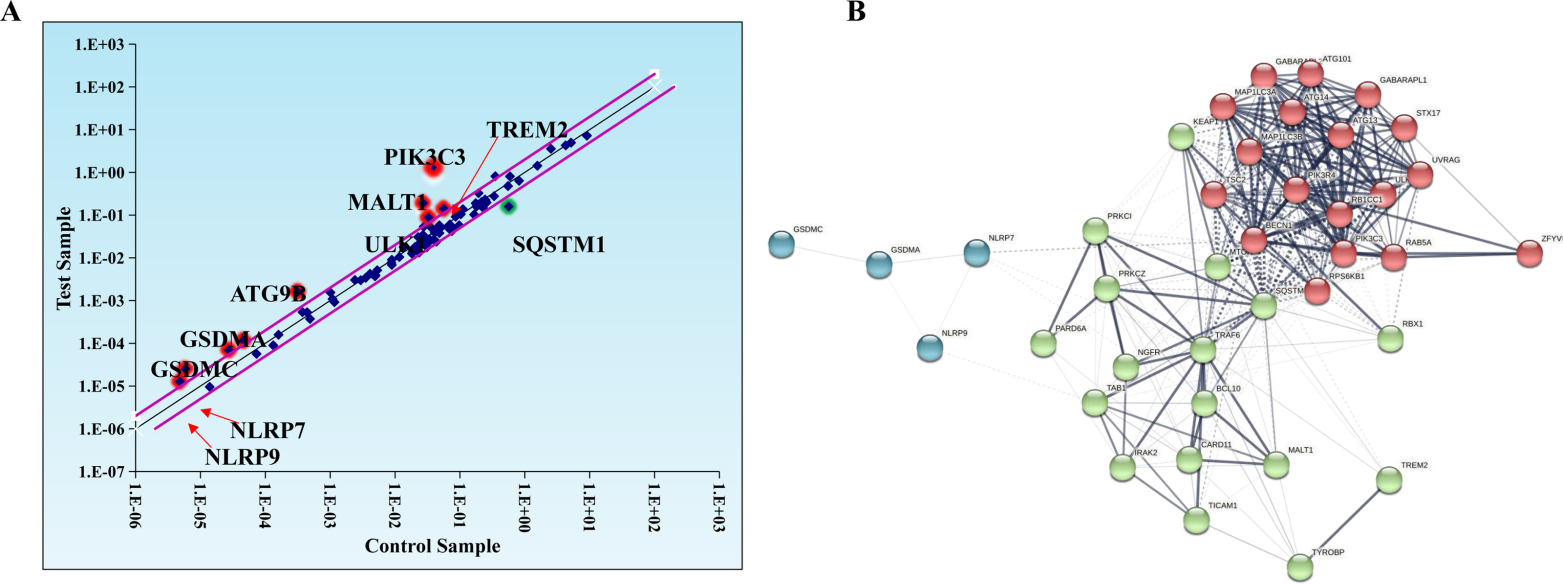
Macrophages incubated by breast cancer cell-derived exosomes induced by ferroptosis regulate autophagy. (A) Scatter distribution of differentially expressed genes screened by autophagy PCR chip. Autophagy PCR chip of PMA-induced THP-1 cells incubated with NC or ferroptosis-induced MDA-MB-231 cell derived-exosomes was used to detect the molecules affecting the polarization of macrophages. (B) Network diagram of protein interaction. The protein–protein network was constructed by Cytoscape V3.6.0.

Evidence suggests that autophagy interacts with ferroptosis (Zhou et al., 2019). Studies have shown that autophagy regulates macrophage M2 polarization (Xu et al., 2021a). Therefore, we investigated whether autophagy is involved in the regulation of macrophage M2 polarization by ferroptosis-induced exosomes. We used autophagy PCR array to analyze the expression of autophagy-related genes in macrophages incubated by exosomes and normal macrophages. It was discovered that a total of 84 autophagy-related genes including TREM2, PIK3C3, MALT1, SQSTM1, ULK1, ATG9B, GSDMA, GSDMC, NLRP7, NLRP9,and SATA3 were screened and there were differences in autophagy-related gene expression between the two groups (Table 1, Fig. 6A). In addition, to construct the gene network regulated by exosomes, we analyzed protein interactions of genes regulated by these exosomes. We discovered that SQSTM1 interacted with PRKC1, and NLRP9 interacted with NLRP7 (Fig. 6B). These results indicated that ferroptosis-induced cancer cell-derived exosomes might inhibited M2 polarization of macrophage by regulating autophagy.

**Table 1:** PCR array results.

Symbol	AVG ΔCt (Ct(GOI) - Ave Ct (HKG))		2ˆ-ΔCt		Fold change	T-TEST
	jushi-exo	jushi	jushi-exo	jushi	jushi-exo/jushi	*p* value
AIM2	6.234293	5.412329	0.013283	0.023482	0.565671	N/A
AKT1	2.298273	2.210982	0.203306	0.215987	0.941288	N/A
AMBRA1	−2.85048	−3.18056	7.212418	9.066582	0.795495	N/A
APIP	4.045998	3.838593	0.060539	0.069899	0.866094	N/A
ATG10	8.055256	7.655842	0.003759	0.004959	0.758166	N/A
ATG12	4.557619	3.731169	0.042464	0.075302	0.563915	N/A
ATG16L1	5.025686	5.45888	0.030699	0.022736	1.350219	N/A
ATG16L2	5.049683	5.135149	0.030192	0.028455	1.061031	N/A
ATG3	4.109241	3.36332	0.057942	0.097172	0.596287	N/A
ATG4A	11.39158	10.9985	0.000372	0.000489	0.761497	N/A
ATG4B	5.183901	4.891058	0.02751	0.033701	0.816292	N/A
ATG4C	5.537392	5.340992	0.021532	0.024672	0.872726	N/A
ATG4D	6.312746	5.778761	0.01258	0.018215	0.690645	N/A
ATG5	4.28616	4.384301	0.051255	0.047884	1.070393	N/A
ATG7	3.220678	3.285738	0.10727	0.10254	1.046128	N/A
ATG9A	4.41106	3.850447	0.047004	0.069327	0.678014	N/A
ATG9B	9.278057	11.62868	0.001611	0.000316	5.100443	N/A
BCL2	7.028812	6.833214	0.007658	0.00877	0.873211	N/A
BECN1	4.718174	4.37468	0.037992	0.048205	0.78813	N/A
BNIP3	2.86507	3.16914	0.137255	0.111172	1.234622	N/A
CAMP	14.10896	13.73799	5.66E−05	7.32E−05	0.773259	N/A
CASP1	12.62139	12.61311	0.000159	0.00016	0.994279	N/A
CASP3	5.633549	5.382243	0.020143	0.023976	0.840135	N/A
CASP4	4.083857	3.825282	0.058971	0.070547	0.835913	N/A
CASP5	10.09031	9.747326	0.000917	0.001163	0.788406	N/A
CASP6	8.195742	8.147902	0.003411	0.003526	0.967384	N/A
CPTP	9.844576	9.84623	0.001088	0.001086	1.001147	N/A
CTSB	−1.83317	−1.33769	3.563196	2.527456	1.409795	N/A
DAPK1	4.693199	4.610878	0.038655	0.040925	0.944537	N/A
DHX9	2.342525	1.96064	0.197165	0.256914	0.767434	N/A
DRAM1	4.402851	3.70068	0.047273	0.07691	0.614647	N/A
DRAM2	3.259428	3.279814	0.104427	0.102962	1.014231	N/A
EEF2K	5.164339	5.189841	0.027886	0.027397	1.017834	N/A
ELAVL1	2.742435	2.145005	0.149432	0.226094	0.66093	N/A
FOXO3	1.617001	2.369759	0.326013	0.193478	1.685011	N/A
GABARAP	2.114115	2.032904	0.230987	0.244363	0.945264	N/A
GABARAPL1	5.23605	4.655978	0.026533	0.039665	0.66893	N/A
GABARAPL2	4.156729	3.866427	0.056066	0.068563	0.817731	N/A
GBP1	4.864159	5.210671	0.034335	0.027004	1.271483	N/A
GJA1	8.364298	8.698523	0.003035	0.002407	1.2607	N/A
GLMN	6.579201	6.42562	0.010458	0.011633	0.899016	N/A
GSDMA	16.26084	17.68252	1.27E−05	4.75E−06	2.678971	N/A
GSDMB	8.381172	8.40025	0.002999	0.00296	1.013311	N/A
GSDMC	13.08052	14.41543	0.000115	4.58E−05	2.522594	N/A
GSDMD	5.277618	5.146923	0.02578	0.028224	0.913391	N/A
HDAC6	5.390301	4.527045	0.023843	0.043373	0.54971	N/A
HMGB1	1.064194	0.860663	0.47824	0.550699	0.868423	N/A
IL18	5.688345	5.505883	0.019393	0.022007	0.881198	N/A
IL1B	0.312044	0.760699	0.8055	0.59021	1.364767	N/A
IRGM	9.337461	9.955019	0.001546	0.001007	1.534276	N/A
LAMP1	2.41256	2.02022	0.187822	0.246521	0.761893	N/A
MALT1	2.402975	5.212597	0.189074	0.026968	7.01101	N/A
MAP1LC3A	5.807266	5.624138	0.017858	0.020275	0.880791	N/A
MAP1LC3B	4.118	4.374613	0.057592	0.048207	1.194671	N/A
MAPK14	2.652147	2.476077	0.159083	0.179733	0.88511	N/A
MAPK8	7.990568	7.653014	0.003932	0.004968	0.791382	N/A
MTOR	5.328228	4.85887	0.024891	0.034462	0.722286	N/A
NAIP	7.880659	7.925061	0.004243	0.004115	1.031255	N/A
NLRC4	5.271233	4.93771	0.025894	0.032629	0.793597	N/A
NLRP1	13.44779	12.88085	8.95E−05	0.000133	0.675047	N/A
NLRP13	16.67346	16.1368	9.57E−06	1.39E−05	0.689361	N/A
NLRP3	7.608006	7.555594	0.005126	0.005315	0.964323	N/A
NLRP7	13.82213	15.16331	6.9E−05	2.73E−05	2.533587	N/A
NLRP9	15.257	17.38283	2.55E−05	5.85E−06	4.364541	N/A
P2RX7	6.793722	6.808134	0.009013	0.008924	1.01004	N/A
PARP1	1.852243	1.573258	0.276961	0.336049	0.82417	N/A
PIK3C3	−0.36532	4.668993	1.288168	0.039309	32.77024	N/A
PIK3CG	3.446569	3.542149	0.091723	0.085843	1.068495	N/A
PIK3R4	5.825134	5.012909	0.017638	0.030972	0.569503	N/A
PRKAA1	2.151386	2.011971	0.225096	0.247934	0.907887	N/A
PYCARD	4.306746	4.305199	0.050529	0.050583	0.998929	N/A
RB1	2.490545	2.355866	0.177939	0.19535	0.910872	N/A
SQSTM1	2.640472	0.837483	0.160376	0.559619	0.28658	N/A
STAT3	3.280954	2.595961	0.102881	0.165401	0.622009	N/A
TLR2	2.397871	2.514836	0.189744	0.174968	1.084451	N/A
TMEM74	10.86252	11.38362	0.000537	0.000374	1.43505	N/A
TNF	5.803364	6.209263	0.017907	0.013515	1.324915	N/A
TP53	8.076166	7.674323	0.003705	0.004896	0.75689	N/A
TP63	10.87344	11.14507	0.000533	0.000442	1.207177	N/A
TREM2	2.834921	4.161452	0.140153	0.055883	2.507989	N/A
ULK1	3.518421	4.938477	0.087267	0.032611	2.675959	N/A
ULK2	7.194036	6.811541	0.006829	0.008903	0.76711	N/A
VIM	0.300192	1.508477	0.812144	0.351482	2.310628	N/A
WIPI1	4.226492	5.177794	0.053419	0.027627	1.933617	N/A
ACTB	−2.09935	−2.09378	4.285174	4.268651	1.003871	N/A
B2M	−2.30737	−2.36687	4.949797	5.158203	0.959597	N/A
GAPDH	0.654741	0.292036	0.635189	0.816748	0.777705	N/A
HPRT1	4.266668	4.812083	0.051952	0.035597	1.459439	N/A
RPLP0	−0.51469	−0.64347	1.428684	1.562082	0.914602	N/A

### Macrophages incubated with ferroptosis-induced exosomes inhibit breast cancer cell progression

**Figure 7:**
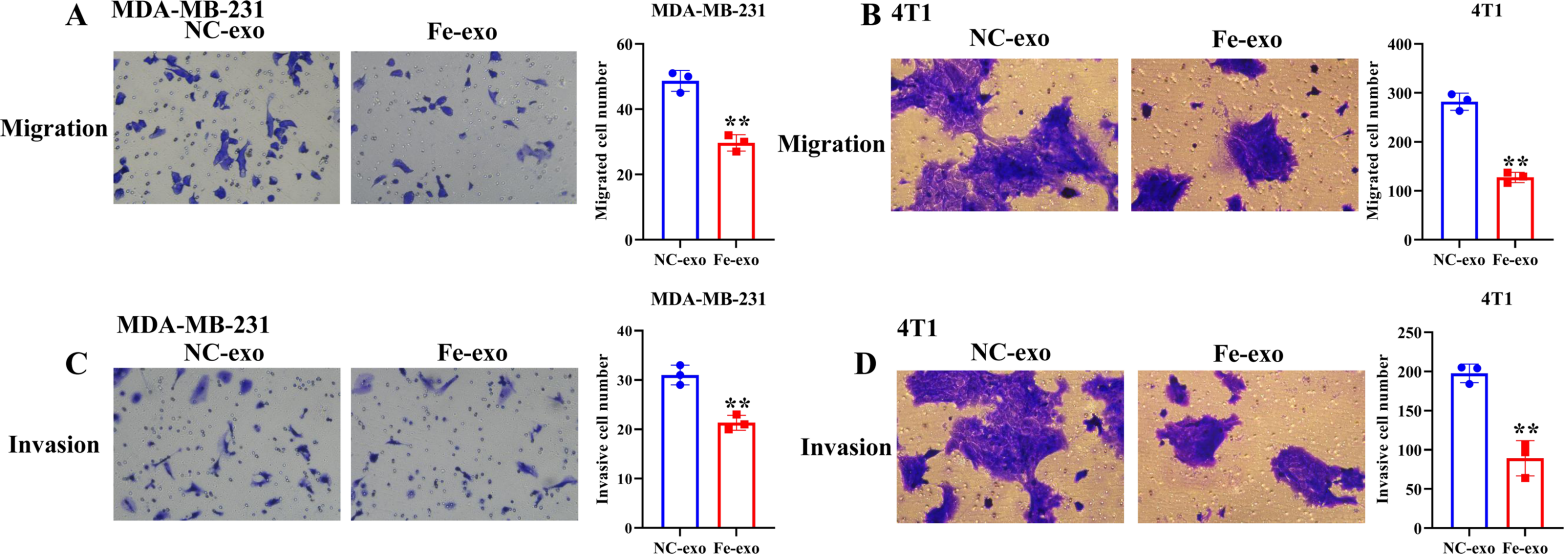
Macrophages incubated by breast cancer cell-derived exosomes induced by ferroptosis suppress breast cancer cell migration and invasion. (A) Cell migration ability of MDA-MB-231 cells incubated by supernatants of PMA-induced THP-1 cells treated with NC or Fe-induced MDA-MB-231 cell derived-exosomes was determined by transwell migration assay. (B) Cell migration ability of 4T1 cells incubated by supernatants of RAW 264.7 cells treated with NC or Fe-induced 4T1 cell derived-exosomes was determined by transwell migration assay. (C) Cell migration ability of MDA-MB-231 cells incubated by supernatants of PMA-induced THP-1 cells treated with NC or Fe-induced MDA-MB-231 cell derived-exosomes was determined by transwell matrigel invasion assay. (D) Cell migration ability of 4T1 cells incubated by supernatants of RAW 264.7 cells treated with NC or Fe-induced 4T1 cell derived-exosomes was determined by transwell matrigel invasion assay. NC-exo represents negative control breast cancer cell-derived exosomes. Fe-exo represents ferroptosis-induced breast cancer cell-derived exosomes. Data are presented as the mean ± SD. ** *p* < 0.01.

To investigate the effect of macrophages incubated with ferroptosis-mediated cancer cell exosomes on cell phenotype of breast cancer, culture supernatants of PMA-induced THP-1 cells and RAW 264.7 cells treated with MDA-MB-231 cell and 4T1 cell-derived exosomes induced by ferroptosis were used to incubate MDA-MB-231 cells and 4T1 cells. Transwell assay showed that PMA-induced THP-1 cells and RAW 264.7 cells incubated by ferroptosis-induced cancer cell-derived exosomes significantly suppressed the migration and invasion of MDA-MB-231 cells and 4T1 cells compared with PMA-induced THP-1 cells and RAW 264.7 cells incubated by control exosomes (Figs. 7A–7D). Collectively, ferroptosis-induced breast cancer cell-derived exosomes inhibited M2 macrophage polarization, which further inhibited breast cancer cell migration and invasion (Fig. S2).

## Discussion

Breast cancer is one of the malignant tumors threatening women’s health (Cruceriu et al., 2020). Recent studies have suggested that ferroptosis are involved in the development of breast cancer (Qiao et al., 2020; Zhang et al., 2021). Nevertheless, the function of ferroptosis-induced exosomes in macrophage polarization and breast cancer progression remains largely unclear. In this study, we found that breast cancer cell-derived exosomes induced by ferroptosis were internalized by macrophages and suppressed M2 macrophage polarization. Furthermore, we confirmed that macrophages incubated by breast cancer cell-derived exosomes induced by ferroptosis inhibited the ability of migration and invasion of breast cancer cells.

Exosomes are nanoscale vesicles that act as an important bridge between cells and between cells and the microenvironment for material exchange and signal transduction (Pegtel & Gould, 2019). Tumor-derived exosomes can be taken up by macrophages in the tumor microenvironment and then participate in tumor progression and metastasis (Baig et al., 2020). Bladder cancer derived exosomes induced macrophage M2 polarization to facilitate bladder cancer progression (Jiang et al., 2021). Macrophages were reported to exhibit M1 and M2 subtypes. M1 macrophages release abundant IL-1β, IL-6, CD86 to play a pro-inflammatory role (Smith et al., 2016). In contrast, M2 macrophages are characterized by increased expression of CD206, Arg-1, and IL-13, which play a regulatory role in the regression of inflammatory response (Roszer, 2015). In our study, breast cancer cell-derived exosomes induced by ferroptosis decreased the expression of Arg1, indicating that they inhibited M2 macrophage polarization. Recently, several studies reported that ferroptosis is associated with immune microenvironment, and ferroptosis participated in regulating immune cells, including T cells, monocytes and macrophages (Kim et al., 2022; Xu, Min & Wang, 2021b). Zhao et al. (2021) discovered that the induction of ferroptosis remarkably decrease the ratio of M2 macrophages in tumor microenvironment in headneck squamous cell carcinoma. Xu et al. (2022) revealed that MXRA8 knockdown repressed M2 macrophage infiltration, whereas this effect was rescued by ferrostatin-1, a ferroptosis inhibitor. Tang et al. (2021a) uncovered that interference of RRM2 inhibited M2 macrophage polarization, while ferrostatin-1 reversed this result. Moreover, evidence has shown that M2 macrophage could facilitate the migration and invasion of cancer cells (Yin et al., 2016). Zhao et al. (2020) found that colorectal cancer cell-derived exosome carried miR-934 to promote M2 polarization of macrophage, which further accelerated liver metastasis of colorectal cancer. Wang et al. (2021) discovered that hepatocellular carcinoma cell-derived exosomes with hsa_circ_0074854 knockdown inhibited M2 polarization of macrophage, which in turn inhibiting hepatocellular carcinoma cell migration and invasion. Similarly, in our study, breast cancer cell-derived exosomes induced by ferroptosis inhibited M2 macrophage polarization, which further suppressed migration and invasion of breast cancer cells.

Autophagy was reported to be closely related with the development of breast cancer (Romero et al., 2019). Ferroptosis can affect cell processes *via* regulating autophagy (Hou et al., 2016). The link between autophagy and ferroptosis is a new trend in recent studies. Amentoflavone repressed cell viability and resulted in cell death through inducing ferroptosis in glioma in autophagy-dependent manner (Chen et al., 2020). Curcumin triggered ferroptosis through inducing autophagy in non-small-cell lung cancer (Tang et al., 2021b). In the present study, we discovered 84 autophagy-related genes in macrophages incubated by breast cancer cell-derived exosomes induced by ferroptosis, including NLRP7 and STAT3. A recent study showed that NLRP7 accelerated tumor progression and promote M2 macrophage polarization in colorectal cancer (Li et al., 2021). Another study discovered that overexpression of STAT3 induced M2 macrophage polarization and stemness in ovarian cancer cells (Ning et al., 2018). Therefore, breast cancer cell-derived exosomes induced by ferroptosis might modulate breast cancer progression *via* mediating autophagy-regulated molecules.

In conclusion, breast cancer cell-derived exosomes induced by ferroptosis were taken up by macrophages and suppressed M2 macrophage polarization, which inhibited migration and invasion of breast cancer cells. These studies provide evidence for the role of breast cancer cell-derived exosomes induced by ferroptosis in macrophage polarization, which then offers new directions for the treatment of breast cancer.

##  Supplemental Information

10.7717/peerj.15060/supp-1 Supplemental Information 1 Flow cytometric analysis of CD86-positive cells and CD206-positive cells in PMA-induced THP-1 cells and RAW 264.7 cells (A) Flow cytometric analysis of CD86-positive cells. (B) Flow cytometric analysis of CD206-positive cells. The cell population with right size was circled in accordance with the forward-scatter (FSC) and side scatter (SSC).

10.7717/peerj.15060/supp-2 Supplemental Information 2 The mechanistic illustration of function of breast cancer cell-derived exosomes in breast cancer

10.7717/peerj.15060/supp-3 Supplemental Information 3 Primer information used in this study

10.7717/peerj.15060/supp-4 Supplemental Information 4 Uncropped gels/blots and raw data

## Additional Information and Declarations

### Data Availability

The following information was supplied regarding data availability: The raw measurements are available in the Supplementary Files.
