# Supplementary figures and images for: Ferroptosis-dependent breast cancer cell-derived exosomes inhibit migration and invasion of breast cancer cells by suppressing M2 macrophage polarization
Source: PeerJ. 2023 Mar 17;11:e15060. doi: 10.7717/peerj.15060 (PMC10026718; doi:10.7717/peerj.15060)

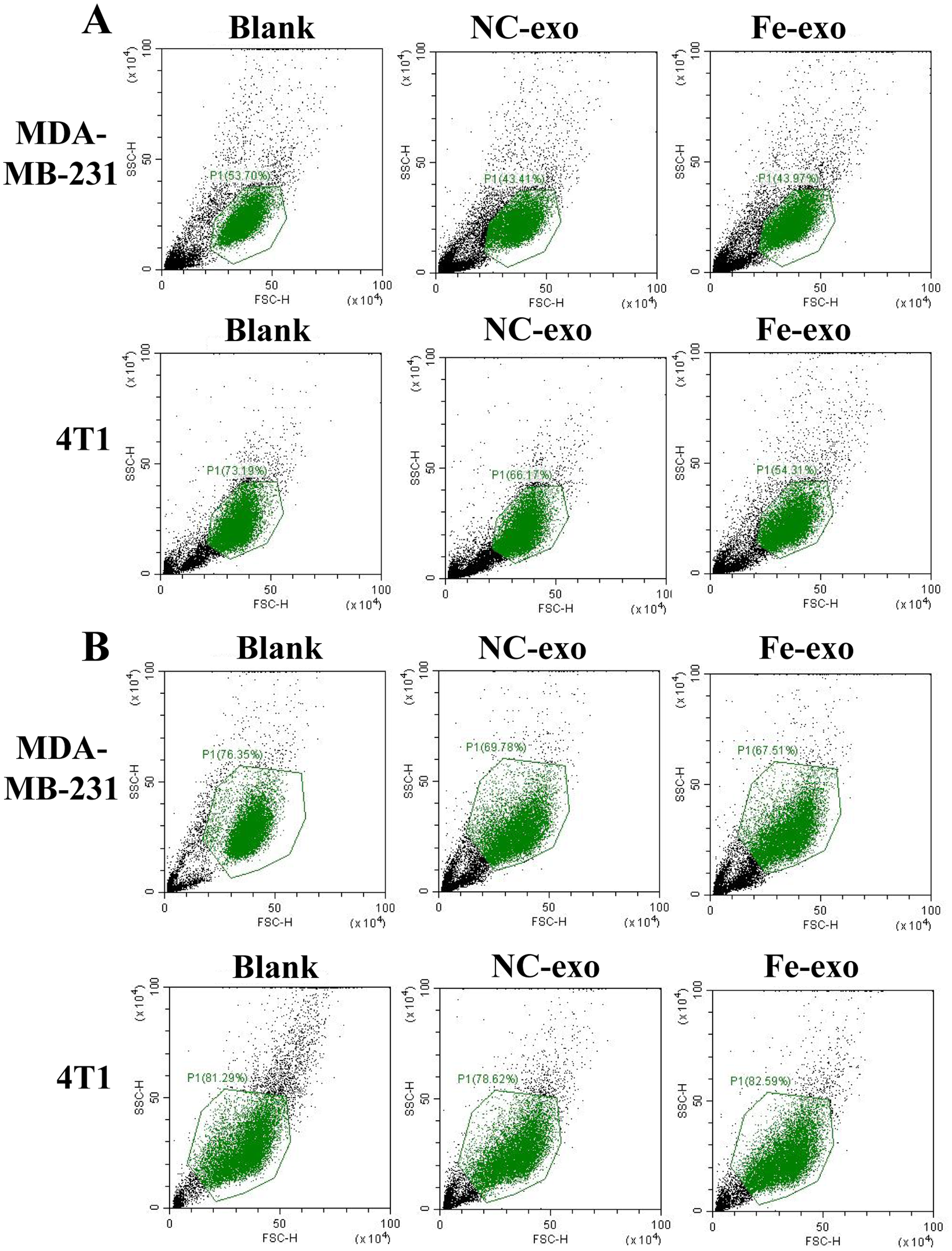

Supplement: Supplemental Information 1 — (A) Flow cytometric analysis of CD86-positive cells. (B) Flow cytometric analysis of CD206-positive cells. The cell population with right size was circled in accordance with the forward-scatter (FSC) and side scatter (SSC). [file peerj-11-15060-s001.png]

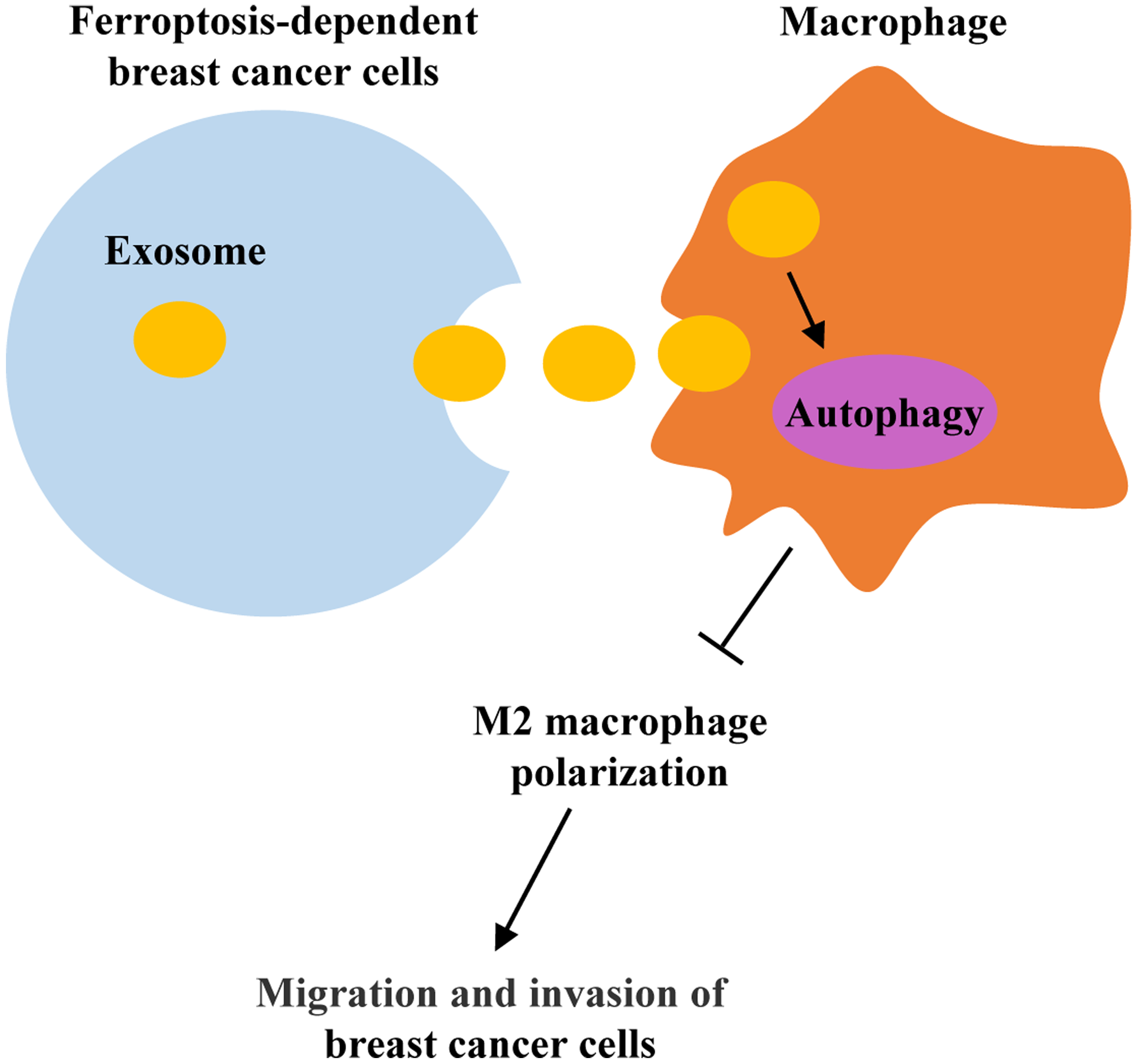

Supplement: Supplemental Information 2 [file peerj-11-15060-s002.png]
