# Supplementary material for: Ferroptosis-dependent breast cancer cell-derived exosomes inhibit migration and invasion of breast cancer cells by suppressing M2 macrophage polarization
Source: PeerJ. 2023 Mar 17;11:e15060. doi: 10.7717/peerj.15060 (PMC10026718; doi:10.7717/peerj.15060)
Supplement: Supplemental Information 3 [file peerj-11-15060-s003.docx]

**Table** **S1 Primers information used in this study.**

| Name | Sequences (5'to3') |
| --- | --- |
| human GAPDH-F | TGACTTCAACAGCGACACCCA |
| human GAPDH-R | CACCCTGTTGCTGTAGCCAAA |
| human Arg-1-F | TGGACAGACTAGGAATTGGCA |
| human Arg-1-R | CCAGTCCGTCAACATCAAAACT |
| human CD206-F | AGGGATCGGGTTTATGGAGC |
| human CD206-R | GAACGGGAATGCACAGGTTG |
| human CD163-F | GAAGACAGAGACAGCGGCTT |
| human CD163-R | GGTATCTTAAAGGCTCACTGGGT |
| human iNOS-F | AGGGACAAGCCTACCCCTC |
| human iNOS-R | CTCATCTCCCGTCAGTTGGT |
| human IL1β-F | GGCCCTAAACAGATGAAGTGC |
| human IL1β-R | TCGGAGATTCGTAGCTGGAT |
| m-GAPDH-F | CAAAATGGTGAAGGTCGGTGT |
| m-GAPDH-R | GAGGTCAATGAAGGGGTCGTT |
| mmu-Arg1-F | TTGGGTGGATGCTCACACTG |
| mmu-Arg1-R | GTACACGATGTCTTTGGCAGA |
| mmu-CD206-F | GCTTCCGTCACCCTGTATGC |
| mmu-CD206-R | TCATCCGTGGTTCCATAGACC |
| mmu-CD163-F | CTGGCGGGTGGTGAAAACA |
| mmu-CD163-R | CAGCCGTTACTGCACACTG |
| mmu-IL1β-F | TTCAGGCAGGCAGTATCACTC |
| mmu-IL1β-R | GAAGGTCCACGGGAAAGACAC |
| mmu-iNOS-F | GGAGTGACGGCAAACATGACT |
| mmu-iNOS-R | TCGATGCACAACTGGGTGAAC |
